# Bilateral facial nerve palsy responded to immunosuppressive therapy in a patient with eosinophilic granulomatosis with polyangiitis

**DOI:** 10.1093/rap/rkac073

**Published:** 2022-08-29

**Authors:** Ai Yorishima, Yusuke Yoshida, Yuta Nanao, Naoya Oka, Sho Masuda, Tomohiro Sugimoto, Shintaro Hirata

**Affiliations:** Department of Clinical Immunology and Rheumatology, Hiroshima University Hospital, Hiroshima, Hiroshima, Japan; Department of Rheumatology, Hiroshima Prefectural Hospital, Hiroshima, Hiroshima, Japan; Department of Clinical Immunology and Rheumatology, Hiroshima University Hospital, Hiroshima, Hiroshima, Japan; Postgraduate Clinical Training Center, Hiroshima University Hospital, Hiroshima, Hiroshima, Japan; Department of Clinical Immunology and Rheumatology, Hiroshima University Hospital, Hiroshima, Hiroshima, Japan; Department of Clinical Immunology and Rheumatology, Hiroshima University Hospital, Hiroshima, Hiroshima, Japan; Department of Rheumatology, Hiroshima Prefectural Hospital, Hiroshima, Hiroshima, Japan; Department of Clinical Immunology and Rheumatology, Hiroshima University Hospital, Hiroshima, Hiroshima, Japan; Department of Clinical Immunology and Rheumatology, Hiroshima University Hospital, Hiroshima, Hiroshima, Japan

Key message

- IVIG and CYC may ameliorate intractable bilateral facial nerve palsy in eosinophilic granulomatosis with polyangiitis.

Dear Editor, Peripheral neuropathy is a common complication of eosinophilic granulomatosis with polyangiitis (EGPA), with a prevalence rate of 58–86%, while that of cranial nerve involvement is only 3–14% [1]. Among the complications of cranial neuropathy in patients with EGPA, facial nerve palsy is second only to ischaemic optic neuropathy [2]. However, to date, bilateral facial nerve palsy attributable to EGPA has not been reported. Here, we present the first report of a patient with EGPA complicated by bilateral facial nerve palsy who responded well to a combination therapy of IVIG and CYC in addition to glucocorticoids.

An 81-year-old woman with a 4-year history of otitis media had been suffering from a dry cough for 4 months. She was diagnosed with bronchial asthma 3 months earlier based on a high concentration of exhaled nitric oxide (141 parts per billion). On admission, a physical examination revealed sporadic purpura on the trunk and extremities and numbness in the peripheral lesions of the lower limbs. Marked eosinophilia (3010/μL), proteinuria and pleural effusion were detected upon a general and systemic examination. Renal biopsy specimens showed the infiltration of lymphocytes and eosinophils into the interstitium, with formation of granulomas.

EGPA was diagnosed according to the Lanham criteria and the criteria of the Ministry of Health, Labour and Welfare of Japan for EGPA [3]. The patient tested negative for ANCAs against MPO and PR3 and the FIP1-like-1-platelet-derived growth factor receptor-α fusion gene. On the 5th day after admission, the patient complained of spilling food from the left corner of her mouth. Physical examination revealed that the left nasolabial fold was slightly shallower than the right (Fig. 1A). On the 6th day, the shallow left nasolabial fold became obvious, suggesting left facial nerve palsy (Fig. 1B). Brain MRI showed no apparent lesion to explain the cause of the palsy, and the serological test for varicella-zoster virus was negative. Thus, we diagnosed facial nerve palsy with cranial neuropathy attributable to EGPA and initiated treatment with 32 mg of methylprednisolone daily (equivalent dose of 1 mg/kg of prednisolone).

**Figure 1.**
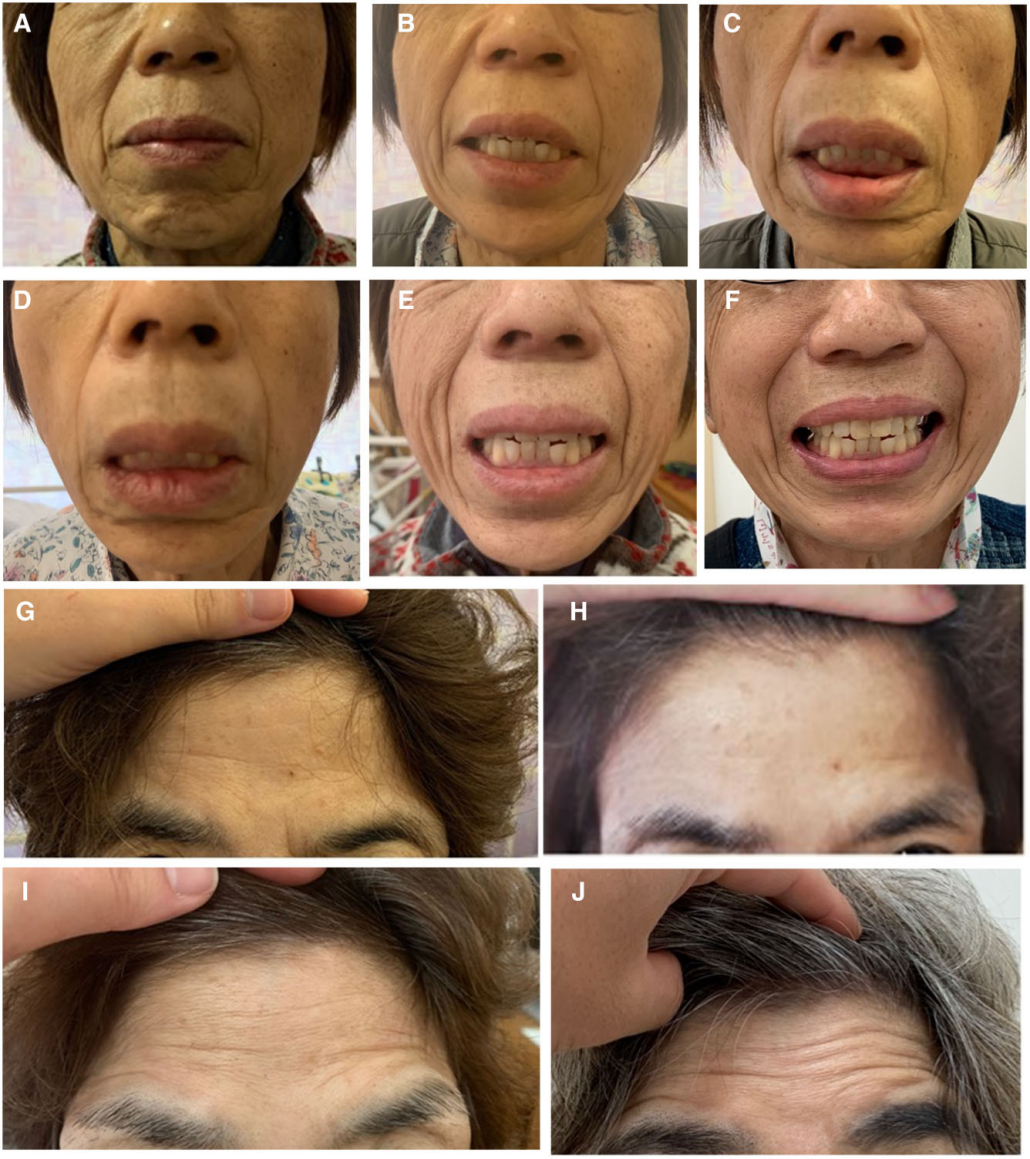
The patient’s clinical course of bilateral facial nerve palsy. **(A, B)** The shallowed left nasolabial fold was observed at rest on the 5th day after admission (A) and when grinning on the 6th day (B). **(C–E)** The shallowed bilateral nasolabial folds when grinning on the 8th (C), 18th (D) and 33rd (E) days after admission. (F) The improved nasolabial fold abnormalities when grinning 6 months after admission. **(G–J)** The forehead wrinkling, observed on the 8th (G), 24th (H) and 33rd (I) days after admission, improved 6 months after admission (J)

On the 8th day after admission, the patient developed ptosis of both corners of her mouth (Fig. 1C) and had difficulty wrinkling her forehead on both sides (Fig. 1G). Methylprednisolone pulse therapy (500 mg daily) was administered from the 9th to the 11th day, while the bilateral paralysis did not improve (Fig. 1D and H). Monthly i.v. CYC (10 mg/kg) was started on the 20th day, and the paralysis tended to improve (Fig. 1E and I). IVIG (400 mg/kg) was administered from the 34th to the 38th day. Six months after admission, she was in good condition, with 7 mg of methylprednisolone daily, and had recovered from the paralysis (Fig. 1F and J).

The mechanisms of neuropathy attributable to EGPA are divided into two categories: ANCA-related fibrinoid necrosis caused by vasculitis and endoneurial infiltration of eosinophils [4]. It is believed that the patient showed symmetrical neuropathies based on the latter aetiology, although we could not clarify why she developed bilateral facial nerve palsy. There have been a few reports of granulomatosis with polyangiitis, an ANCA-associated vasculitis with bilateral facial nerve palsy [5]. It is suggested that patients with ANCA-associated vasculitis with ear involvement, including otitis media, tend to develop facial nerve palsy [6].

It was helpful to evaluate the state of facial nerve palsy to assess the face at rest, during wrinkling of the forehead and when grinning. The recognition of changes in the patient’s clinical course helped us to determine the intensity of the immunosuppressive treatment administered. We added CYC to high-dose glucocorticoids according to the 2021 ACR/Vasculitis Foundation guidelines because of the severity of facial nerve palsy, which is considered an organ-threatening manifestation [7]. In addition, we administered IVIG concurrently based on a randomized controlled trial that demonstrated the improvement of residual peripheral neuropathy attributable to EGPA [8].

In conclusion, this is the first report of a patient with EGPA complicated by bilateral facial nerve palsy. The patient’s clinical course indicated that early recognition of facial nerve palsy and proper treatment with combined glucocorticoids, CYC and IVIG might be successful.

## Data Availability

Data are available upon reasonable request by any qualified researchers who engage in rigorous, independent scientific research, and will be provided following review and approval of a research proposal and Statistical Analysis Plan (SAP) and execution of a Data Sharing Agreement (DSA). All data relevant to the study are included in the article.

## References

[1] Wolf J , Bergner R, Mutallib S, Buggle F, Grau AJ. Neurologic complications of Churg–Strauss syndrome – a prospective monocentric study. Eur J Neurol 2010;17:582–8. 20050889 10.1111/j.1468-1331.2009.02902.x

[2] André R , Cottin V, Saraux JL et al; French Vasculitis Study Group (FVSG). Central nervous system involvement in eosinophilic granulomatosis with polyangiitis (Churg-Strauss): report of 26 patients and review of the literature. Autoimmun Rev 2017;16:963–9. 28709761 10.1016/j.autrev.2017.07.007

[3] Lanham JG , Elkon KB, Pusey CD, Hughes GR. Systemic vasculitis with asthma and eosinophilia: a clinical approach to the Churg-Strauss syndrome. Medicine (Baltim) 1984;63:65–81. 10.1097/00005792-198403000-00001 6366453

[4] Oka N , Kawasaki T, Matsui M et al Two subtypes of Churg-Strauss syndrome with neuropathy: the roles of eosinophils and ANCA. Mod Rheumatol 2011;21:290–5. 21188447 10.1007/s10165-010-0400-9

[5] Jeong SM , Park JH, Lee JI et al Progressive bilateral facial palsy as a manifestation of granulomatosis with polyangiitis: a case report. Ann Rehabil Med 2016;40:734–40. 27606281 10.5535/arm.2016.40.4.734 PMC5012986

[6] Harabuchi Y , Kishibe K, Tateyama K et al Clinical features and treatment outcomes of otitis media with antineutrophil cytoplasmic antibody (ANCA)-associated vasculitis (OMAAV): a retrospective analysis of 235 patients from a nationwide survey in Japan. Mod Rheumatol 2017;27:87–94. 27166750 10.1080/14397595.2016.1177926

[7] Chung SA , Langford CA, Maz M et al 2021 American College of Rheumatology/Vasculitis Foundation Guideline for the management of antineutrophil cytoplasmic antibody-associated vasculitis. Arthritis Care Res (Hoboken) 2021;73:1088–105. 34235880 10.1002/acr.24634 PMC12344527

[8] Koike H , Akiyama K, Saito T, Sobue G; Research Group for IVIg for EGPA/CSS in Japan. Intravenous immunoglobulin for chronic residual peripheral neuropathy in eosinophilic granulomatosis with polyangiitis (Churg-Strauss syndrome): a multicenter, double-blind trial. J Neurol 2015;262:752–9. 25577176 10.1007/s00415-014-7618-y PMC4363522

